# Distinct age-associated molecular profiles in acute myeloid leukemia defined by comprehensive clinical genomic profiling

**DOI:** 10.18632/oncotarget.25443

**Published:** 2018-05-29

**Authors:** Katherine Tarlock, Shan Zhong, Yuting He, Rhonda Ries, Eric Severson, Mark Bailey, Samantha Morley, Sohail Balasubramanian, Rachel Erlich, Doron Lipson, Geoff A. Otto, Jo-Anne Vergillo, E. Anders Kolb, Jeffrey S. Ross, Tariq Mughal, Philip J. Stephens, Vincent Miller, Soheil Meshinchi, Jie He

**Affiliations:** ^1^ Department of Hematology/Oncology, Seattle Children’s Hospital, Seattle WA, USA; ^2^ Clinical Research Division, Fred Hutchinson Cancer Research Center, Seattle WA, USA; ^3^ Foundation Medicine, Cambridge MA, USA; ^4^ Nemours Center for Cancer and Blood Disorders, Nemours-Alfred I. DuPont Hospital for Children, Wilmington DE, USA; ^5^ Tufts University Medical Center, Boston MA, USA

**Keywords:** acute myeloid leuekmia, sequencing, pediatric, adult, genomic profiling

## Abstract

Large scale comprehensive genomic profiling (CGP) has led to an improved understanding of oncogenic mutations in acute myeloid leukemia (AML), as well as identification of alterations that can serve as targets for potential therapeutic intervention. We sought to gain insight into age-associated variants in AML through comparison of extensive DNA and RNA-based GP results from pediatric and adult AML. Sequencing of 932 AML specimens (179 pediatric (age 0–18), 753 adult (age ≥ 19)) from diagnostic, relapsed, and refractory times points was performed. Comprehensive DNA (405 genes) and RNA (265) sequencing to identify a variety of structural and short variants was performed. We found that structural variants were highly prevalent in the pediatric cohort compared to the adult cohort (57% vs. 30%; *p* < 0.001), with certain structural variants detected only in the pediatric cohort. Fusions were the most common structural variant and were highly prevalent in AML in very young children occurring in 68% of children < 2 years of age. We observed an inverse trend in the prevalence of fusions compared to the average number of mutations per patient. In contrast to pediatric AML, adult AML was marked by short variants and multiple mutations per patient. Mutations that were common in adult AML were much less common in the adolescent and young adult cohort and were rare or absent in the pediatric cohort. Clinical CGP demonstrates the biologic differences in pediatric vs. adult AML that have significant therapeutic impacts on prognosis, therapeutic allocation, disease monitoring, and the use of more targeted therapies.

## INTRODUCTION

The complex genomic landscape of acute myeloid leukemia (AML), with several recurrent cytogenetic and molecular abnormalities, is an important factor in understanding the variable outcomes achieved in patients [1–3]. Large scale next generation sequencing (NGS) efforts including the Therapeutically Applicable Research to Generative Effective Treatments (TARGET) initiative and The Cancer Genome Atlas (TCGA) have made significant contributions to our understanding of the complexity of the genomic landscape of AML [4–6]. Certain NGS technologies enable comprehensive and simultaneous interrogation of the AML genome and transcriptome. Sequencing of DNA and RNA can allow for identification of a variety of alterations, including base pair substitutions, insertions and deletions, copy number alterations, and gene fusions.

Comprehensive genomic profiling (CGP), that utilizes DNA and RNA sequencing, has led to an improved understanding of oncogenic mutations in AML, as well as alterations that can serve as targets for potential therapeutic intervention. Increased appreciation of the heterogeneity of AML indicates that there are significant biologic differences between pediatric and adult AML [7, 8]. Some of the more recently detected mutations in AML have much higher frequencies in adults compared to children, and this can have significant clinical implications as targeted agents are developed against these mutations [9–11].

Sequencing technologies have advanced rapidly over the past few years and are now routinely incorporated in clinical care for some tumors, with results available to physicians in approximately 2–4 weeks in some cases [12]. The FoundationOne Heme (F1H) platform integrates data from targeted DNA and RNA profiling, thus increasing the breadth and improved sensitivity to identify rearrangements which result in aberrant expression of fusion transcripts. Targeted RNA capture sequencing is used to identify a spectrum of fusion genes with known roles in malignant transformation and therapeutic response, and to identify novel fusions with likely diagnostic and therapeutic relevance. Genomic profiling allows for refined risk stratification, as variants not detected with conventional genomic testing are defining previously unrecognized subsets of patients with specific genomic and epigenomic alterations. It is critical to identify molecular subgroups of patients who experience poor outcomes with current treatments. Refined risk stratification can lead to enhanced prognostication for patients and families, but also identifies patients with the most to benefit from alternative therapeutic approaches, such as early intervention with novel treatments in therapeutic clinical trials or intensification of therapy with hematopoietic stem cell transplant (HCT). Sequencing efforts in AML are essential to improve understanding of age-dependent drivers of leukemogenesis, and can assist in the identification and prioritization of distinct therapeutic strategies for all age groups. We sought to gain insight into age-associated variants in AML through comparison of extensive DNA and RNA-based GP results from pediatric and adult AML.

## RESULTS

A total of 932 patient specific specimens from diagnostic, relapse, or refractory time points underwent CGP. Disease status was known for 515 (55%) and unknown for 417 (45%) samples. For samples with known disease status, 247 were classified as diagnostic and 268 were classified as relapsed/refractory (Supplementary Table 2). According to the 2 age defined cohorts, *n* = 179 patients (range 0–18 years) were classified as pediatric and *n* = 753 (range 19–87 years) were classified as adult. Given the broad range in ages across the cohorts, especially the adult cohort, for purposes of further analysis we subdivided the cohorts to evaluate patients ages 16–40 years, defined as adolescent and young adult (AYA) cohort.

Among the pediatric cohort, 171 (96%) specimens had ≥1 variant detected and in the adult cohort, 737 (98%) had ≥1 variant detected (Supplementary Table 3). Overall, a total of 571 genomic variants of all types involving 131 genes were detected in pediatric cohort and 3,018 variants involving 219 genes in the adult cohort.

**Figure 1:**
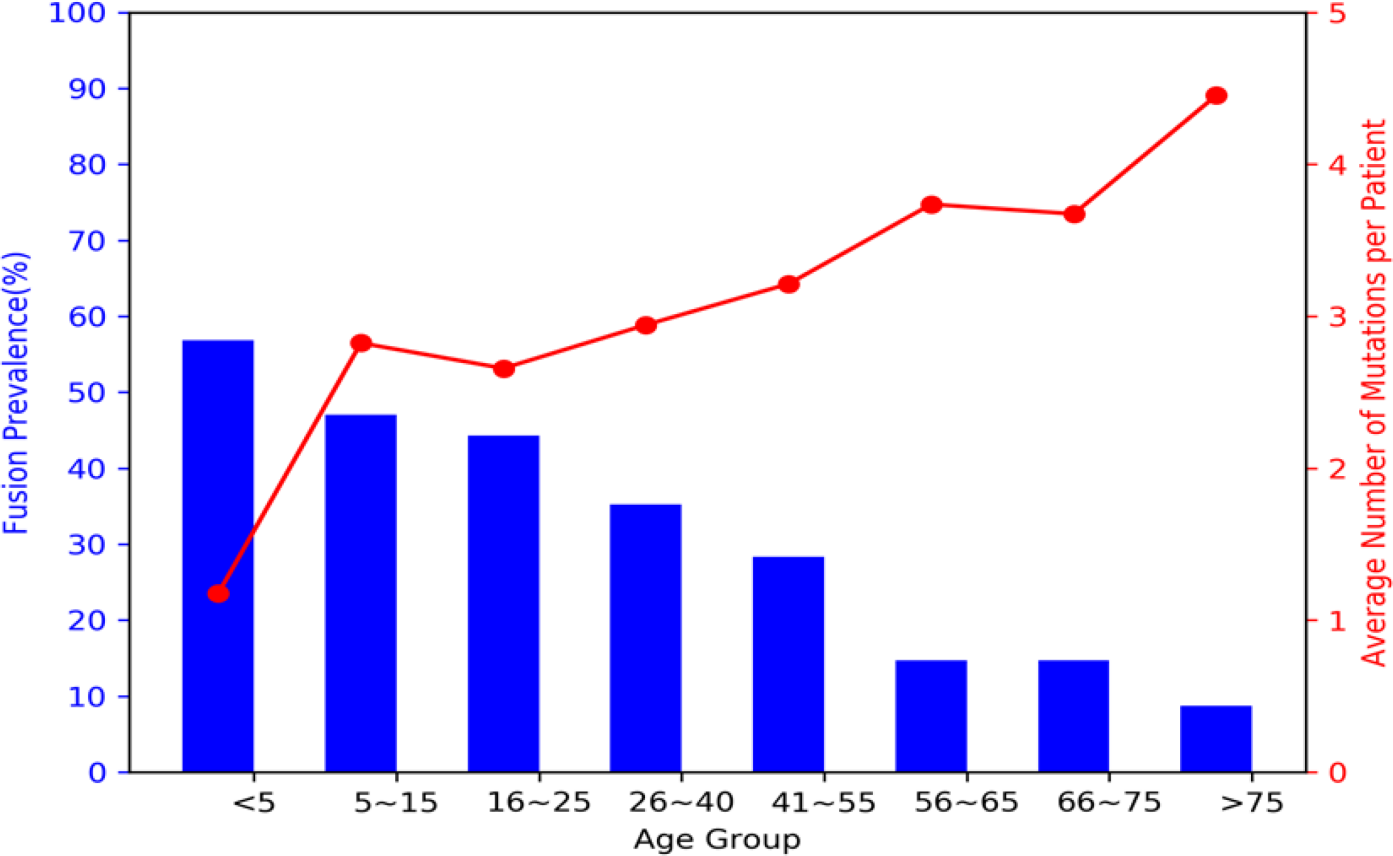
Prevalence of fusions vs. average number of mutations per patient according to age Fusions (blue bars) were highly prevalent in the pediatric population, especially they young children with AML, and decreased with age. In contrast, the average number of mutations per patient (red line) was inversely related to the fusion prevalence and increased with age.

Mutations in the pediatric and adult age groups were further analyzed according to the type of genomic variant. Structural variants were highly prevalent in pediatric AML with a prevalence of 57 % vs. 30 % in adult AML (*p <* 0.001), with fusions being the most prevalent structural variant detected. We further analyzed the prevalence of structural variants using more specific age defined cutoffs. Fusions were the most prevalent in infant AML (<2 years) with a prevalence of 68% and decreased to 54% in children 2–10 years of age and continued to decrease with age to a prevalence of 9% in patients >75 years of age (Figure 1). We observed a strong inverse trend in the prevalence of fusions contrasted to the average number of mutations per patient. Infants had an average of 2 genomic variants/patient and this increased with age to an average of 4.4 in patients > 75 years of age (Figure 1).

**Figure 2:**
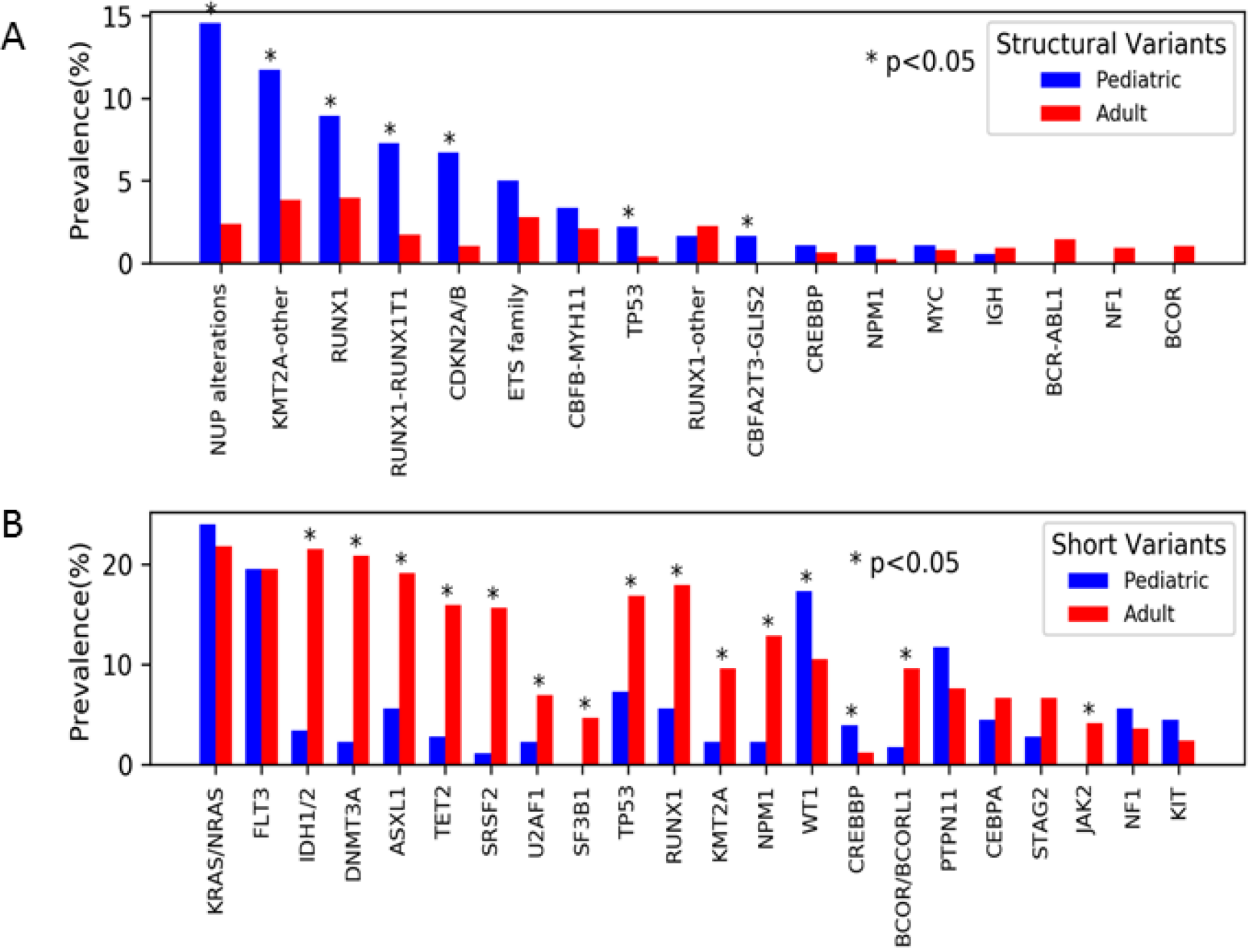
Prevalence of genomic variants by age (**A**) Structural variants were overall more prevalent among the pediatric (blue) vs. adult (red) cohort. The most common structural variants included those involving NUP genes (namely NUP98 and NUP214), KMT2A, and RUNXI (specifically RUNX1-RUNX1T1). (**B**) Short variants were overall more prevalent among adults.

The genes most commonly altered by structural variants were analyzed according to prevalence among the pediatric vs. adult cohort. Significant differences in prevalence were observed between the pediatric vs. adult cohort for structural variants involving the *NUP* family of genes (14.5% vs. 2.4%; *p <* 0.001), *KMT2A* (11.7% vs. 3.8%; *p <* 0.001), *RUNX1* (8.9% vs. 4.0; *p <* 0.01), *CDKN2A/B* (6.7 vs 1.1%; *p <* 0.001) (Figure 2A). A variety of *NUP* alterations occurred in the pediatric cohort. The most common *NUP* variant was *NUP98-NSD1* (*n* = 12) accounting for 44% of all *NUP* variants, other detected variants included *NUP98-KDM5A* (*n* = 7, 26%) *DEK-NUP214* (*n* = 2, 7%), *NUP214-SET* (*n* = 2, 7%), *NUP98-BRWD3* (*n* = 1, 4%), *NUP98-TAF3* (*n* = 1, 4%), and losses of *NUP98* (*n* = 2, 7%). In the adult cohort, fusions involving *NUP98-NSD1* (*n* = 9, 50%) were the most common *NUP* alterations, followed by *DEK-NUP214* (*n* = 3; 17%). Alterations of *CDKN2A/B* (pediatrics *n* = 12, adults *n* = 8) involved *CDKN2A* deletions in all cases, and in 75% (*n* = 15, pediatric = 8, adult = 7) of cases co-occurred with *CDKN2B* deletions. Fusions and deletions of *TP53* were observed in both cohorts, but were more prevalent in the pediatric cohort (2.2% vs 0.4%; *p =* 0.03). Fusions involving *CBFA2T3-GLIS2* were exclusively seen the pediatric AML with a prevalence of 2% (Figure 2A).

Short variants were analyzed in the 2 cohorts, and in contrast to structural variants we observed that the prevalence of short variants increased with age, with a prevalence of 68% in infant AML compared to 98% in patients > 70 years of age. Although there were some commonly mutated genes in both cohorts, including *NRAS*, *KRAS*, and *FLT3*, there were significant differences in prevalence according to age. Genes involved in epigenetic modification (*DNMT3A*, *ASXL1*, and *TET2*) were highly prevalent in the adult cohort (*n* = 345, 45.8%), but much less common in the pediatric cohort (*n* = 18, 10.1%; *p <* 0.01; Figure 2B). Mutations involved in RNA splicing (*SRSF2*, *U2AF1*, *SF3B1*) were also significantly more common in the adult cohort (*n* = 204, 27.1%) and were rare or absent in the pediatric cohort (*n* = 6, 3.4%; *p <* 0.01; Figure 2B). The age breakdown of these mutations was further analyzed and we found they occurred mostly commonly in older AML, decreased in prevalence in AYA AML, and were exceedingly rare in patients ≤ 18 years. Adults ≥ 60 years accounted for 85% (*n* = 152) of *TET2* (*n* = 177), 75% (*n* = 120) of *ASXL1* (*n* = 159), and 62% (*n* = 108) of *DNMT3A* (*n* = 173) mutations. A similar age distribution was also observed in genes involved in RNA splicing, with 87% (*n* = 104) of *SRSF2* (*n* = 120), 68% (*n* = 42) *U2AF1* (*n* = 62), and 69% (*n* = 24) of *SF3B1* (*n* = 35) mutations occurring in patients ≥60 years of age.

Short variants in *TP53* were significantly more prevalent in the adult cohort compared to the pediatric cohort (16.9% vs. 7.3%; *p <* 0.001) as were *RUNX1* (17.9% vs. 5.6%; *p <* 0.001). Mutations in *KMT2A,* primarily partial tandem duplications (PTDs), were also more prevalent in adult vs. pediatric cohort, 9.6% vs 2.2% respectively (*p <* 0.001), as were those involving *BCOR/BCORL1* with a prevalence of 9.6% vs. 1.7% respectively (*p <* 0.001; Figure 2B). *WT1* short variants were detected in 17.3% of adults vs. 10.5% of pediatric samples (*p =* 0.014). The higher prevalence in the adult cohort of short variant mutations involving *TP53*, *KMT2A*, and *RUNX1* is in contrast to the higher prevalence of fusions involving these same genes in the pediatric cohort. Recurrent short variants involving *GATA1* (*n* = 4) and *JAK1* (*n* = 4) were exclusive to the pediatric cohort. Additionally, short variants involving *MEN1*, *AKT1*, *CDC73*, *WHSC1*, *GSK3B*, *MYCN*, *FGF7*, *FGF6*, *FGFR1*, *TAF1*, *SMAD2*, *MAP3K14* (*n* = 1 each) were detected exclusively in the pediatric cohort.

**Figure 3:**
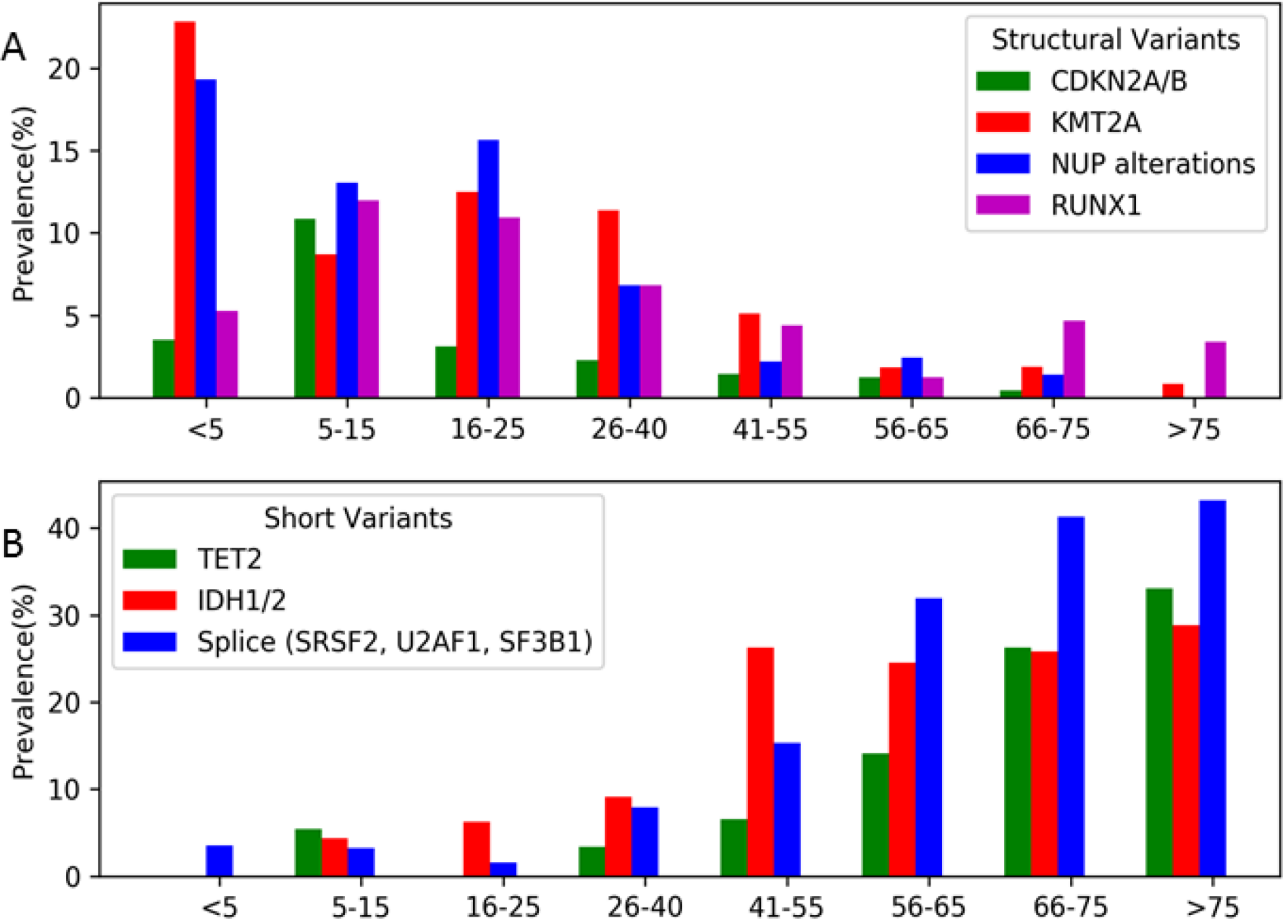
Prevalence of the selected common structural and short variants according to age subsets (**A**) Selected common structural variants (*CDKN2A/B*, *KMT2A*, *NUP* and *RUNX1*) were highly prevent in young patients and exceedingly rare in older patients (> 65 years), however AYA patients (age 16-40) demonstrated a profile unique both end of the age spectrum, but had a higher prevalence of structural variants than adult and older adult AML. (**B**) Selected common short variants (*TET2*, *IDH1/2*, *SRSF2*, *U2AF1*, and *SF3B1*) were rare or not detected in young patients and highly prevalent in older patients. Similarly to structural variants, AYA patients demonstrated a profile unique both end of the age spectrum, but many of the most commonly detected short variants detected in adult AML were rare in the AYA cohort, demonstrating that targeted agents for these alterations will have a low impact in the AYA cohort overall.

Given the heterogeneity of AML, especially among the distinct age groups, we further analyzed the genomic landscape of AML in AYA patients. Distinct differences in this age group were observed when compared to either spectrum of the pediatric and adult cohorts. Structural variants were detected at a higher prevalence in the AYA cohort compared to patients > 40 years in *KMT2A* (11% vs. 2.1%), *CDKN2A* (2% vs. 0.8%), *NUP* (7% vs. 1.3%), and *RUNX1* (7% vs. 3.3%; Figure 3A). Mutations in some of the most commonly mutated genes in adult AML (*TET2*, *ASXL1*, *DNMT3A*, *IDH1/2*) were much less common or rare, occurring with a prevalence of <10%, in AYA patients (Figure 3B). *TET2* mutations were detected in 3% of AYA samples vs. 30% in older adults (Figure 3B), and importantly were absent in the adolescent subset of patients (16–25 years; *n* = 64). Among young adult patients (26–40 years of age, *n* = 88), the prevalence of mutations in *IDH1/2* was 9% (*n* = 8), and was 6% (*n* = 4) in adolescent patients, much lower than the ≈25% observed in the older adult cohorts. Among genes involved in RNA splicing, the prevalence in young adults and adolescents was 8% (*n* = 7) and 2% (*n* = 1) respectively, again much lower than the 30–40% detected in older adults.

We analyzed the prevalence of the different types of alterations among the 515 patients with known disease status. In general, many mutations had similar prevalence among patients with newly diagnosed vs. relapsed/refractory AML. However, we did detect that mutations in *DNMT3A* and *IDH2* were enriched in the relapsed/refractory samples (*p =* 0.02 and *p =* 0.05) respectively. In contrast, mutations in *ASXL1* were more prevalent among newly diagnosed patients (*p =* 0.04), and a strong trend for a higher prevalence of *TET2* mutations among this cohort as well (*p =* 0.055). Overall, there were similar prevalence in most mutations across the 2 disease cohorts (data not shown).

### Co-occurring mutations

**Figure 4:**
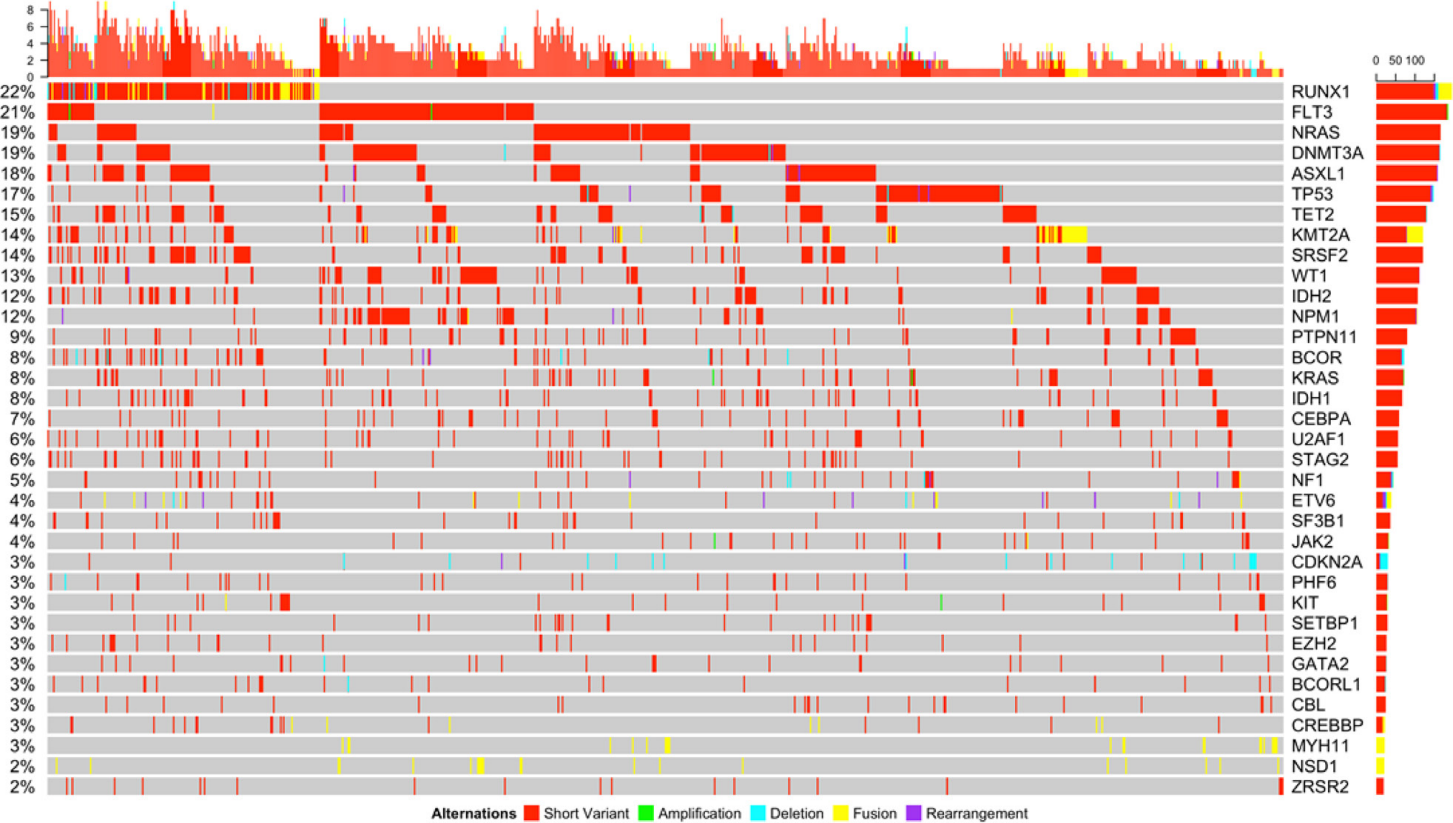
Somatic genomic alterations of all types (short variants, amplifications, deletions, fusions, and rearrangements) with a frequency of ≥2% and associated co-occurring mutations detected in the entire cohort

Multiple variants per sample were detected frequently throughout the entire cohort across the mutational spectrum (Figure 4). The average number of variants of all kinds per patient was 3.19 (range 0–10) vs. 4 (range 0–11) in the pediatric vs. adult cohort respectively (*p* < 0.001). We further analyzed the co-occurrence data for some of the most commonly involved genes according to the type of variant and found strong patterns of co-occurrence between the different types of variants. Strong tendencies to co-occur were detected in the commonly observed co-occurring mutations such as *RUNX1* translocations (namely t(8;21) and *CBFB/MYH11* inv(16)) and *KIT* mutations (*p =* 0.005 and *p =* 0.03 respectively; Supplementary Table 4). Strong tendencies to co-occur were also noted between *CDKN2A* variants and *MLLT10* structural arrangements (*p =* 0.03). We also noted a tendency to occur among splicing mutations and short variants in *RUNX1*, *ASXL1*, *STAG2*, *SETBP1*, and *TET2* (*p <* 0.001, *p <* 0.001, *p =* 0.001, *p =* 0.038, *p <* 0.001; Supplementary Table 4). Among the transcription factors, we found a strong tendency towards co-occurrence in a few mutations involving these genes such as *CEBPA* with *IKZF1* and *GATA2* (*p =* 0.02 and *p =* 0.03 respectively).

We detected strong tendencies towards mutual exclusivity among the mutations. The splicing mutations were not seen with a number of the structural variants, including *KMT2A*, *NUP98-DEK214*, *CBFB-MYH11* (*p =* 0.006, *p <* 0.001, and *p =* 0.03 respectively; Supplementary Table 4). Splicing mutation alterations were also not associated with *TP53*, *IDH1/2* and *WT1* mutations (*p <* 0.001, *p =* 0.04, *p =* 0.004 respectively; Supplementary Table 4). *NPM1* mutations were strongly associated with *FLT3* and *DNMT3A* mutations (*p <* 0.001 and *p <* 0.001 respectively), however they tended to be mutually exclusive with *ASXL1*, *RUNX1* short and structural variants, *KMT2A* short variants, *BCOR/BCORL1*, and *TP53* mutations (*p <* 0.001, *p <* 0.001, *p =* 0.03, *p =* 0.004, *p =* 0.004, and *p =* 0.02 respectively; Supplementary Table 4). In general, we noted that *TP53* mutations had no common cooperating mutations, but tended to be mutually exclusive of many of the mutations analyzed (Supplementary Table 4).

### Variant allele frequency

**Figure 5:**
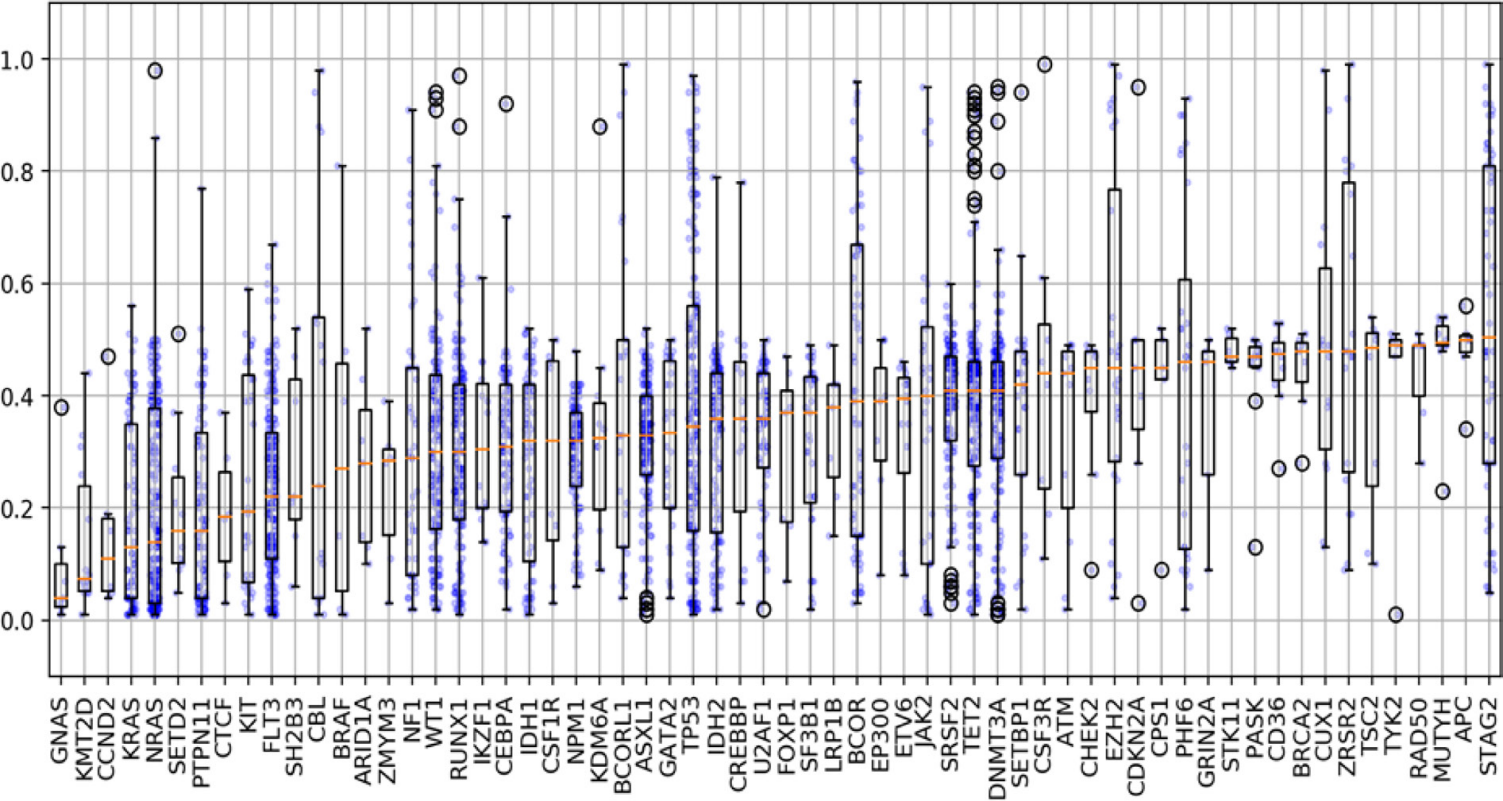
Variant allele frequencies for associated selected genomic alterations Evaluation of the differences in variant allele frequency (VAF) that occurred among short variants (SNVs, indels) and found a broad range of VAFs among all mutations tested (range 1–100%). Several mutations demonstrated VAFs well below the median (ex: NRAS/KRAS) suggesting these mutations are likely subclonal. In contrast, among several genes the VAFs were noted to be well above the median (ex: TP53, BCOR, STAG2), demonstrating that in certain patients these mutations could be clonal or represent other genomic changes. Blue dots represent AF for each mutation with outliers predicted by computational modeling highlighted in black circles.

We evaluated the differences in variant allele frequency (VAF) that occurred among the short variants and found a broad range of VAFs among all mutations tested (1–100%). For many of the mutations analyzed, we found that the VAFs ranged widely among the samples. Our findings demonstrate the heterogeneity and clonality that occurs in AML, and that the significance of a given mutation must be interpreted on an individual patient basis. There were a few mutations that had a median VAF < 0.2 (*NRAS*, *KRAS*, *KMT2D*), suggesting that these mutations are most likely subclonal (Figure 5). However, there were a several mutations that demonstrated VAFs well above the median, demonstrating that in certain patients these mutations may be clonal. Given our findings on many of the mutations with low VAF, which are presumed to be subclonal, we subsequently analyzed the co-occurring mutational profiles eliminating mutations with a VAF ≤ 0.2. We found that in all cases the dominant co-occurring mutational profiles were largely retained, however certain genes such as *NRAS*, *KRAS*, *KIT* or *JAK* were less commonly observed (data not shown). The ability to determine the clonality of a mutation is especially critical in determining which mutations may be an appropriate target for therapy. For many of the genes analyzed, the maximum VAF detected was 0.5. For a number of genes in this cohort (e.g. *SETD2*, *NPM1*, ASXL1, *U2AF1*, *SF3B1*) the mutations were often detected with a VAF of 0.3–0.5 (Figure 5). This finding suggests that alterations in this cohort of genes are heterozygous in all circumstances.

Among all the short variants, a total of 163 mutations were found to have VAFs 0.7, suggesting a copy number alteration (CNA) leading to copy neutral loss of heterozygosity (CN-LOH). VAFs of ≥ 0.7 were noted to be particularly frequent in *TP53* (*n* = 31/174), *STAG2* (*n* = 23/58), *TET2* (*n* = 15/175), *BCOR* (*n* = 16/69), *EZH2* (*n* = 8/28), and *WT1* (*n* = 7/138), *PHF6* (*n* = 6/28), and *ZRSR2* (7/28). Among the 163 mutations with VAF ≥ 0.7, 109 were confirmed to be a result of CN-LOH or single copy deletion of the genomic DNA as predicted by the computational method. *STAG2*, *BCOR, BCORL1, PHF6, and ZRSR2* are located on the X chromosome and among samples with VAFs ≥ 0.7, 100% were male and thus hemizygous for such alterations (Supplementary Figure 1).

The computational method was able to provide unambiguous zygosity prediction for at least one of the mutations detected in a total 297 (*n* = 52 pediatric; adult 245) specimens. We observed that among the adult cohort, 54% (*n* = 133) had at least one mutation predicted under LOH as compared to 31% (*n* = 16; *p <* 0.002) in the pediatric cohort. We further compared the VAFs among patients with newly diagnosed vs. relapsed/refractory AML and found no significant difference overall among the 2 cohorts (Supplementary Figure 2). A slight trend towards a higher frequency of lower VAFs was noted among patients with relapsed/refractory AML (*p =* 0.09). This appeared to be driven by a slightly higher prevalence of VAFs < 0.3 in relapsed/refractory patients as there was no differences observed among mutations with VAFs ≥ 0.3 among the 2 cohorts.

### Orthogonal validation

**Figure 6:**
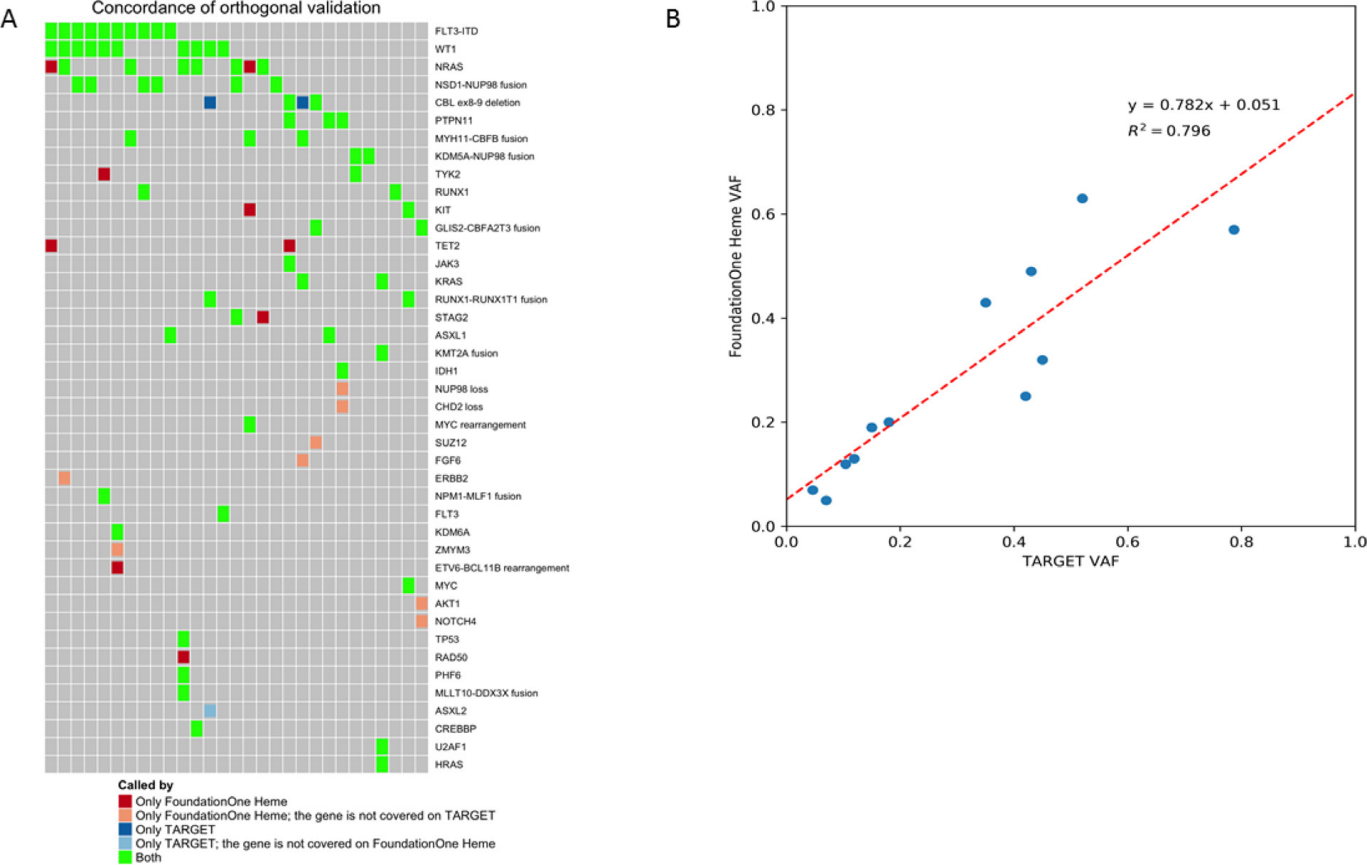
Orthogonal validation between F1H and TARGET platforms (**A**) Among genes covered by both panels, 69/80 (86%) of results were concordant. Among the discordant results (*n* = 9), those identified by F1H only were explained (*n* = 7) by the targeted exome sequencing utilized by TARGET. Among the 2 samples not identified as somatic by F1H, these were whole exon deletions of CBL that were not called as likely somatic by the platform. (**B**) Correlation of VAF results for FLT3/ITD between the 2 platforms demonstrated a high degree of correlation, with the F1H platform able to detect somatic mutations at high sensitivity with regard for allele frequency.

A subset of 30 specimens underwent orthogonal validation to compare the results of F1H to those obtained from clinical sequencing and karyotyping as well as comprehensive NGS on the TARGET Initiative. High rates of concordance were observed between the F1H platform and results obtained from clinical testing. All conventional clinical results were detected by the F1H panel. The F1H panel also was able to detect translocations missed by clinical conventional karyotyping. Further, comparison of results between commonly analyzed genes on the F1H and TARGET panels demonstrated a high degree of concordance. Among genes covered by both panels, 69/80 (86%) of results were concordant (Figure 6A). Among the 11 discordant variants, 9 were detected by the F1H panel only and 2 by the TARGET analysis. Both of the variants missed by F1H were *CBL* exon 8/9 deletions. The TARGET analysis utilized targeted exome sequencing for a subset of samples, thus explaining the 7 samples with variants detected by F1H, where genes were not sequenced in the TARGET samples. Comparison of VAF between the F1H platform and results of TARGET for *FLT3*/ITD mutations was performed. A high degree of correlation was observed for VAF results between the 2 platforms (*r*^2^ = 0.8; Figure 6B), with the F1H platform able to detect somatic mutations at high sensitivity with regard for allele frequency.

### Miscellaneous targeted mutations

Comprehensive sequencing revealed a number of mutations in patients that are not commonly tested for in AML, but are observed in a variety of other malignancies. Further, a number of these mutations can serve as targets for therapeutic interventions especially with small molecular inhibitors. Mutations involving *CSF3R* were detected in pediatric (*n* = 2) and adult (*n* = 8) samples, namely the T618I mutation (*n* = 8) and frameshift mutations resulting in truncation (*n* = 2) which have been found to be amenable to targeting with JAK inhibitors and dasatinib respectively [13]. Further, *BRAF* mutations were identified in 6 samples (pediatric *n* = 2, adult *n* = 4), including *BRAF* V600E (*n* = 2) and other kinase activating mutations, which are commonly seen in melanomas and histiocytic disorders and been highly amenable to BRAF-inhibitor therapy [14, 15].

## DISCUSSION

Extensive clinical sequencing highlighted key differences between the genomic landscapes of pediatric vs. adult AML, and as well as the distinct biologic age group of AYA patients. Our results confirmed previous large-scale sequencing initiatives demonstrating that AML is a very heterogeneous disease with multiple distinct oncogenic drivers and many patients harboring multiple mutations, suggesting distinct mechanisms of cooperation [4, 16–18]. This is the largest study to directly compare pediatric to adult AML specimens using comprehensive NGS sequencing.

We demonstrate that pediatric AML is characterized by frequent structural alterations, which were highly prevalent among the youngest patients. This is in line with what has been reported, with a variety of recently identified cryptic fusions not identified by conventional cytogenetics present in a majority of infants with AML [19, 20]. Importantly, these events can have prognostic and therapeutic implications that are important to clinicians and patients, as patients with high-risk lesions may be appropriate for intensification with HCT or the introduction of novel therapies [20, 21]. Many of the fusions detected by this clinical assay would not have been detected by conventional karyotyping. Increased recognition of genomic events that are not detected with conventional diagnostic technologies is critical to providing information on their prognostic significance. Although incorporation of a few cytogenetic and molecular features have provided a successful risk-based therapeutic strategy in AML, [1, 3, 22], further refinement according to the genomic profile of a patient’s leukemia can allow for optimal therapy allocation. Identification of patients with high risk genomic features will prioritize those with very poor outcomes with current therapies for early intervention with therapeutic intensification and upfront introduction of novel therapies [23].

We observed a strong inverse correlation between the prevalence of fusions and the number of mutations per patient, which is line with the results of the TARGET sequencing initiative as reported by Bolouri *et al.* [6]. For many infants, the structural variant was the only genomic variant detected, whereas older adults harbored multiple mutations. This relationship suggests that the structural variants are strong drivers of leukemogenesis, sometimes the only genomic event detected and at other times occurring in the setting of 1–2 additional mutations. For genes where both structural and short variants were common (ex: *KMT2A*, *RUNX1*, *TP53*), we observed that structural variants were more prevalent in the pediatric cohort while short variants were more prevalent in the adults. This suggests that in many cases while the genes and functions involved in leukemogenesis are similar among across all ages in AML, the mechanisms of genomic alterations differ significantly between the pediatric and adult patients.

We believe that the high prevalence of structural variants, including some that are only identified by DNA and RNA-inclusive sequencing, provides additional information with significant clinical relevance. Many of the structural variants detected, especially the fusions, are strong driver events that are present in the founding clone and can be used for disease detection [24, 25]. As many fusions are stable from diagnosis through relapse, they function as effective markers of disease and can be used for monitoring of measurable residual disease (MRD) [24, 26]. Additionally, MRD monitoring of fusions by PCR can be prognostic, with the time to disappearance or significant decrase as well as level of detection at the time of transplant providing prognostic information and identifying a group of patients with poor outcomes [27, 28]. Patients with continued or emergent levels of MRD are among those with the highest risk for relapse and are most in need of novel therapeutic interventions. The ability to detect low level disease at the time of emergence may allow for more successful therapeutic intervention. Among our dataset, fusions were detected in 57% of the pediatric cohort, this represents a majority of patients where detection of the fusion, especially cryptic fusions, at diagnosis could allow for peripheral blood monitoring for therapeutic response and subsequent need intervention. Although structural variants pose a more significant challenge for therapeutic targeting, current investigative efforts for some oncogenic fusions are aimed at the resultant aberrant epigenetic regulation.

Differences between the 2 age groups in regards to cooperation between the mutated genes were detected. For genes where both structural and short variants were common (ex: *KMT2A* and *RUNX1*), there were a significantly higher number of cooperating events among the short variants compared to structural events. This data further supports the transformative oncogenic capacity of these damaging structural events that are hallmarks of AML in very young patients. Additionally, cooperating mutations differed between the 2 types of variants as additional co-occurring mutations were significantly more common among short variants. Distinct cooperating patterns emerged among some of the most commonly mutated genes, and for some genes co-occurring alterations with just a select few genes were observed in a majority of patients.

Among the pediatric cohort, we identified a number of structural alterations not identified by current conventional karyotyping techniques, highlighting the importance comprehensive sequencing for this group of patients. Alterations identified on the F1H panel included those among *CDKN2A* (p16/Ink4) and *CDKN2B,* which are well established in solid malignancies, including lung, breast, pancreatic, melanoma, and a variety of head and neck carcinomas, as well as in pre-B cell acute lymphoblastic leukemia (ALL) [29–31]. *CDKN2A/B* function as important tumor suppressor genes and inactivation through homozygous deletion has been associated with poor prognosis in a variety of solid malignancies common in adults. Additionally, in some studies of pediatric and adult pre B ALL, poor initial response to therapy and inferior survival were observed in patients with *CDKN2A* deletions [32, 33], but may be augmented with intensification of therapy with allogeneic HCT [34]. Further investigation into association with outcome with AML is needed, as these benefits may benefit from augmented therapy. Among patients with *CDKN2A/B* alterations, mutations of the RAS pathway (*NRAS*, *KRAS*, *PTPN11*) were common and although *RAS* mutations are often subclonal, tyrosine kinase inhibitors against the RAS pathway has shown early clinical activity and may represent a potential pathway for therapeutic targeting in patients where *RAS* pathway mutations occur in the dominant clone [35].

Many of the patients in the AYA age group are treated on protocols designed for the adult age group overall. Our findings in this age cohort demonstrate the distinct biology of AML in younger adults as compared to older adults, which in many cases is due to acquisition of multiple mutations over time. These genomic distinctions underscore the need for therapeutic efforts that are aimed at this cohort of patients. Recent efforts aimed at treating this AYA population distinctly from older adults and very young patients will likely be of benefit to this group of patients. These genomic differences must be taken into account as early as drug development and clinical trial design. For example, *IDH1/2* inhibitors have demonstrated some encouraging results in preclinical and early phase clinical trials, however given the low prevalence of *IDH1/2* mutations in adolescents and young adults it is unlikely that this class of drugs will have any significant impact in treating AML in this group of patients [36]. Therapeutic trials that account for the biologic differences in AML across a wide spectrum of ages will allow for more patient-directed studies, and optimize the use of targeted agents to improve outcomes for patients with AML.

AML is a heterogeneous and clonal disease, and indeed many of the samples in our cohort had multiple mutations detected, including multiple putative therapeutic targets. In this setting, prioritization of mutations is critical to appropriate interpretation of clinical sequencing data, and we posit that VAFs may help further elucidate NGS results. The presence or absence of mutations can be thought of as binary on initial analysis, however in the setting of multiple mutations the VAF can help provide a better understanding of the clinical role of a particular leukemogenic mutation in an individual patient’s leukemia. This can be important to evaluating the clonal nature of the leukemia, but also a mutation’s role as a potential therapeutic target. The VAF represents the relative mutational burden in the sample assuming a relatively pure leukemic sample. Although the VAF is impacted by the amount of disease present in the sample and with very low blast counts may be difficult to determine, all samples in our cohort had ≥20% blasts. In general a higher VAF is considered to a marker of a higher clonal burden, with mutations with lower VAFs generally thought be subclonal or of later acquisition leukemogenesis. Thus, if multiple potentially targetable mutations are present in a sample, then that with the higher VAF may be a more optimal therapeutic target as it is present in the greatest number of leukemia cells. However, as we are just beginning to interpret the clinical application of NGS results, caution is advised in simply interpreting a hierarchical mutational pattern by VAF. It is important to note that VAFs are not only the predictor of a mutation’s role, as many mutations that not are strong driver events may be particularly susceptible to clonal evolution, even if present at a high VAF [37]. Further, chemotherapy can apply strong selection pressures further altering the genomic landscape, with acquisition of additional mutations or in some cases allowing for emergence of a subclone [38].

We had available clinical data regarding disease status for 55% of the cohort, and in an analysis of the differences in VAFs among patients with newly diagnosed vs. relapsed/refractory AML found no significant differences. There was no difference among higher VAFs 0.3, this suggests that mutations present in the bulk of leukemia population are more likely to be clonal and persist at the time of relapse. Among lower VAFs <0.3, we observed a slight trend towards lower frequency in newly diagnosed samples. This may represent the ongoing leukemogenic evolution that occurs following chemotherapy exposure and disease progression where additional subclonal or passenger mutations may be acquired. However, we did not compare diagnostic and relapse samples from the same patient and it is important to note that we and others have previously shown that higher diagnostic VAFs are more likely to predict or persist at relapse, suggesting that these mutations are more likely to indicate presence in the founder clone and are more appropriate for therapeutic targeting [5, 17, 39].

Recognition of CN-LOH was detected with this clinical assay in a majority of samples with high VAFs. Copy neutral-LOH or acquired uniparental disomy is becoming increasing recognized in hematologic malignancies initially with SNP arrays and now with comprehensive sequencing efforts [40]. CN-LOH can occur through a variety of mechanisms and may signify additional disruption to the recombination/DNA repair pathway and can contribute to leukemogenesis [41]. Acquired CN-LOH can lead to either effective knockout (ex: *TP53*) or enhanced expression (ex: *FLT3/ITD*), thus making a significant impact on leukemic progression [41–43]. The clinical significance of CN-LOH in AML is not yet known, however these lesions have been shown to represent therapeutic targets as in JAK2 myeloproliferative neoplasms and there are suggestions that they may be associated with inferior outcomes in pediatric AML [44, 45]. Events involving CN-LOH were observed in pediatric and adult samples in our study, with the adult cohort demonstrating a higher prevalence. However, we acknowledge that this age-associated analysis was limited by the smaller size of each of the cohort and number of genes with appropriate data available for analysis. Further information regarding the prognostic impact of CN-LOH vs single allele variants in AML is needed and our findings suggest that these are events are prevalent, especially among patients with high VAFs. Further, these mutations may identify the lesions that are most appropriate for directed therapy.

Comprehensive sequencing research initiatives in AML highlight the genomic heterogeneity and provided significant insights into the biology of the disease. The genomic information yielded through a number of sequencing efforts in AML has entered the clinical spectrum and has begun to further define risk group and therapeutic strategies. Our findings underscore the importance of DNA and RNA sequencing in AML. Many previously described assays have largely focused on DNA-based approaches, or have used gene-specific RNA tests to detect fusion transcripts in a small set of genes. The F1H approach described here combines DNA and RNA capture to allow for the identification of full-length transcripts driven by promoter rearrangements by DNA-sequencing, as well as fusion transcripts which are more easily identified by RNA profiling. We demonstrate the impact of clinical DNA and RNA-based GP for patients across all age spectrums and highlight the distinct biology of pediatric, AYA, and older adult AML. Although many of the same genes and pathways are altered across all age groups, our findings highlight the differences in the mechanisms of alterations with structural variants with potent leukemogenic impacts dominating the genomic landscape of AML. Clinical GP can provide important information to physicians, patients, and families that can identify molecular alterations that enhance prognostication, disease monitoring, therapeutic allocation, and may provide a target for therapeutic intervention.

## MATERIALS AND METHODS

### Sequencing

Specimens from patients with AML from peripheral blood, bone marrow, or biopsy of extramedullary sites of disease underwent DNA and RNA extraction, with RNA being converted to cDNA. DNA and cDNA underwent library construction and hybridization capture with biotinylated DNA oligonucleotides as previously described using the F1H sequencing platform [12]. Selected libraries were pooled and sequenced on the Illumina HiSeq2500. This approach can identify fusions transcripts present in as few as 10–20% of cells.

A subset of samples from the TARGET cohort were utilized for orthogonal validation, with sequencing methodologies including NGS sequencing, CLIA-certified fragment length analysis, Sanger sequencing or karyotyping, described previously [5, 46–48]. TARGET NGS primarily utilized whole genome or targeted exome capture sequencing as previously described [6]. For validation of the *FLT3*/ITD mutations and corresponding allele frequencies, we compared F1H results to those obtained by clinical *FLT3* mutational testing. PCR amplification and fragment length analysis to determine the presence of an ITD mutation and to calculate the ratio between mutant and wild type *FLT3* to determine the allele frequency was performed as previously described [46, 48].

### Analysis

Overall, 932 samples were sequenced to high depth averaging 569X for DNA (405 genes) and > 3 million unique on-target pairs for RNA (265 genes) on the F1H platform (Supplementary Table 1A, 1B). Base substitutions, indels, copy number alterations and rearrangements were called as previously described [12, 49]. Short variants included single nucleotide variants as well as oligonucleotide insertions and deletions (indels). Structural variants included fusions, rearrangements, and copy number variants (CNVs). A subset of 323 specimens passed strict quality control measures for copy number alteration (CNA) analysis. Samples with transplant history, contamination, non-informative copy number model with undeterminable cause (e.g. low purity or low aneuploidy) were excluded from CNA analysis. Patients were divided into 2 cohorts according to age at the time of sample collection, with patients 0–18 years classified as pediatric and patients ≥ 19 years classified as adult. A subset of samples from the TARGET initiative (*n* = 30) underwent validation by orthogonal methodologies, Wilcoxon rank sum test was used to evaluate differences between two groups of quantitative values. Fisher’s exact test was used to evaluate difference between two proportions. Odds ratio (OR) analysis was used for the co-occurring mutation analysis, with *p*-values determined by Fisher’s exact test. The null hypothesis was that the mutation occurrence in the two genes is independent, and the frequency of the mutation co-occurrence in the two genes is proportional to their uncorrelated occurrence. We used a computational method to predict zygosity status for each mutation [50]. This method does not require a patient matched normal control, but can yield a prediction of ambiguous mutation origin due to certain limitations. Approval for patients included on the F1H sequencing, including a waiver of informed consent and a HIPPA waiver of authorization, was obtained from the Western Institutional Board (WIRB). For patients sequenced as part of the TARGET initiative, informed consent was obtained in accordance with the Declaration of Helsinki and institutional review boards of all participating institutions approved the clinical protocols.

## SUPPLEMENTARY MATERIALS FIGURES AND TABLES












