# Supplementary material for: Distinct age-associated molecular profiles in acute myeloid leukemia defined by comprehensive clinical genomic profiling
Source: Oncotarget. 2018 May 29;9(41):26417–30. doi: 10.18632/oncotarget.25443 (PMC5995178; doi:10.18632/oncotarget.25443)
Supplement: Supplementary file 1 [file oncotarget-09-26417-s001.pdf]

Distinct age-associated molecular profiles in acute myeloid leukemia defined by comprehensive clinical genomic profiling

SUPPLEMENTARY MATERIALS

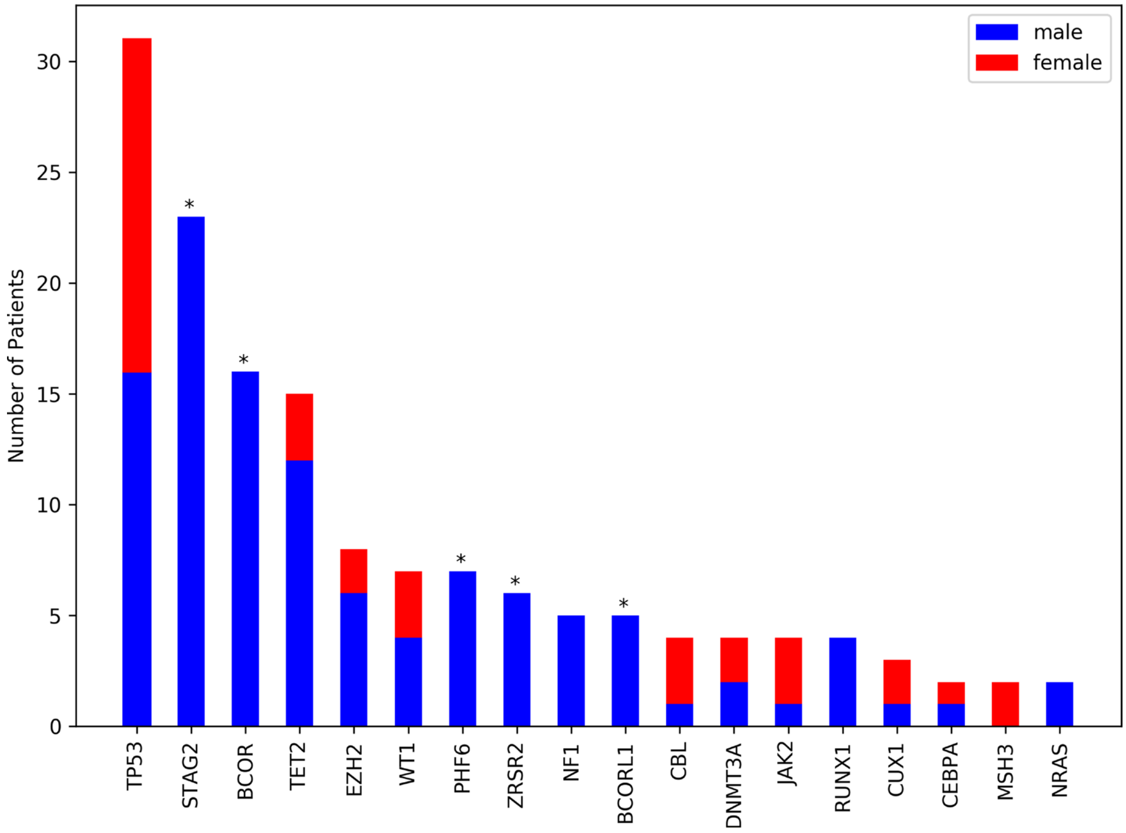

Supplementary Figure 1: Breakdown of samples by gender (males in blue and females in red) among samples with VAF > 0.7, with a *indicating a gene located on X chromosome.

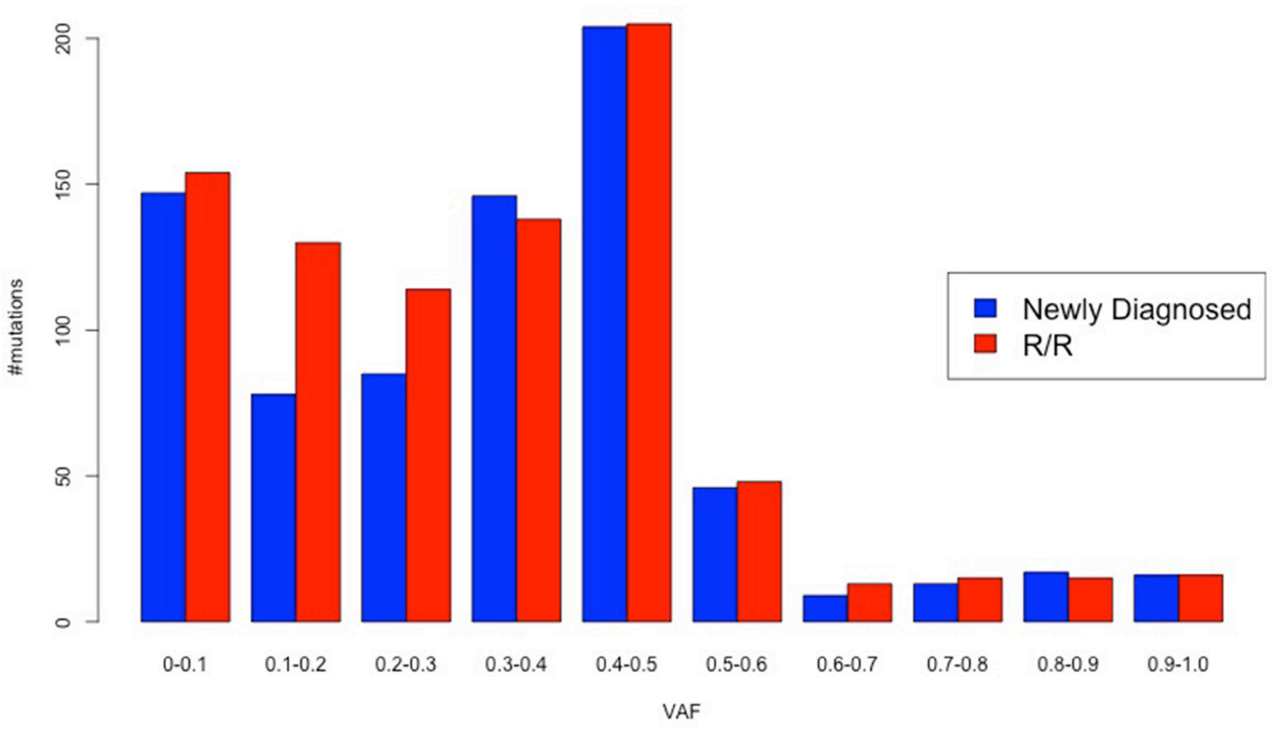

Supplementary Figure 2: Range and occurrence of VAFs in newly diagnosed (blue) and relapsed/refractory (red) samples.

Supplementary Table 1A: Genes targeted in DNA hybridization capture F1H assay. Genes with selected introns targeted for rearrangement detection are listed separately. See Supplementary_Table_1A

Supplementary Table 1B: RNA transcripts targeted for capture in F1H assay. See Supplementary_Table_1B

Supplemental Table 2: Age and diagnostic status for samples included in the cohort. See Supplementary_Table_2

Supplemental Table 3: Genomic annotation of all variants identified in the cohort using the F1H sequencing panel. See Supplementary_Table_3

Supplemental Table 4: Tendencies of co-occurrence among the most commonly identified mutations in the cohort. See Supplementary_Table_4
