# Supplementary material for: Distinct age-associated molecular profiles in acute myeloid leukemia defined by comprehensive clinical genomic profiling
Source: Oncotarget. 2018 May 29;9(41):26417–30. doi: 10.18632/oncotarget.25443 (PMC5995178; doi:10.18632/oncotarget.25443)
Supplement: Supplementary file 2 [file oncotarget-09-26417-s002.docx]

**Supplementary Table 1A: Genes targeted in DNA hybridization capture F1H assay. Genes with selected introns targeted for rearrangement detection are listed separately**

| **Exons** | | | | | | | | |
| --- | --- | --- | --- | --- | --- | --- | --- | --- |
| TP53 | NRAS | KRAS | ASXL1 | TET2 | DNMT3A | CDKN2A | KMT2D (MLL2) | RUNX1 |
| CCND1 | CREBBP | BCL2 | SRSF2 | CDKN2B | RB1 | BRAF | KMT2A (MLL) | FLT3 |
| WHSC1 | ARID1A | IDH2 | SF3B1 | ATM | EZH2 | PTPN11 | JAK2 | CD36 |
| ETV6 | WT1 | STAG2 | MYD88 | U2AF1 | TRAF3 | TNFAIP3 | MYC | CDKN2C |
| NOTCH1 | NF1 | BCOR | B2M | NPM1 | ZRSR2 | TNFRSF14 | BCL6 | IDH1 |
| LRP1B | FGFR3 | EP300 | BIRC3 | PHF6 | CEBPA | SETBP1 | SETD2 | MUTYH |
| ABL1 | FAF1 | PRDM1 | BRCA2 | PASK | ARID2 | BCL7A | KDM6A | IKZF1 |
| FAS | PTEN | CBL | APC | CARD11 | BCORL1 | STAT3 | DDX3X | CUX1 |
| NOTCH2 | GATA2 | CDKN1B | CHEK2 | STK11 | CXCR4 | MAP3K14 | CIITA | FBXW7 |
| MCL1 | AXIN1 | KIT | PIM1 | CD58 | CCND3 | JAK3 | PAX5 | PIK3CA |
| ZMYM3 | CHD2 | BCL10 | TSC2 | TCF3 | ATR | ICK | XPO1 | MSH6 |
| SPEN | TBL1XR1 | SGK1 | CD79B | CSF3R | SMARCA4 | PIK3R1 | NCOR2 | FOXP1 |
| SOCS1 | CKS1B | MALT1 | RHOA | TRAF2 | MAP2K1 | CD70 | FANCA | CRLF2 |
| POT1 | HIST1H1D | ALK | CDH1 | PDCD1LG2 | CPS1 | TP63 | TYK2 | PCLO |
| PBRM1 | IRF4 | RAD50 | ETS1 | GNAS | IKZF3 | IGF1R | BRCA1 | EGFR |
| MTOR | SUZ12 | CD274 | CIC | BRIP1 | MDM4 | EPHB1 | GRIN2A | CTCF |
| TLL2 | KMT2C (MLL3) | JAK1 | RET | CDK6 | KDM4C | TNFRSF11A | PTCH1 | FBXO11 |
| MAP3K6* | FLCN | FOXO1 | SMARCB1 | KDM5C | TMEM30A | ID3 | ZNF703 | CCND2 |
| MEF2B | PALB2 | ATRX | KDM5A | FGF14 | MLH1 | HNF1A | PDGFRA | CSF1R |
| MSH3 | FGFR1 | VHL | KAT6A (MYST3) | FGF3 | TAF1 | MPL | HIST1H1E | PTPRO |
| IL7R | FLT4 | ERBB4 | PTPN6 | RAF1 | FGF6 | FGF4 | FGF19 | PIK3CG |
| SMAD4 | CAD | TCL1A | MDM2 | ROS1 | RELN | FLT1 | NOD1 | CCT6B |
| STAT6 | EED | KDR | C11orf30 | NUP98 | IKBKE | MYCN | MED12 | ERBB2 |
| PC | INPP4B | AKT3 | NTRK1 | SOCS3 | PIK3R2 | BTG2 | HIST1H2AM | HIST1H2AG |
| BAP1 | PDCD11 | MAF | TSC1 | CDK12 | SUFU | HGF | IRF8 | PDGFRB |
| MAP3K1 | NF2 | BRD4 | SRC | KLHL6 | LRRK2 | EPHA7 | FGF10 | HIST1H1C |
| SDHA | AMER1 (FAM123B or WTX) | LEF1 | CTNNB1 | ERG | ARAF | RARA | BARD1 | CDK4 |
| RICTOR | FGF23 | AKT1 | ARHGAP26 | BRSK1 | HIST1H2AC | GNA12 | GNA13 | FHIT |
| MEN1 | CDC73 | NUP93 | GATA3 | GATA1 | ARFRP1 | GTSE1 | SOX10 | SMO |
| JARID2 | TUSC3 | CCNE1 | MEF2C | S1PR2 | FANCC | HIST1H3B | MET | HRAS |
| RAD51* | DDR2 | MAFB | SMC1A | HIST1H2BJ | EPHA3 | TRAF5 | RAD21 | WISP3 |
| SDHC | CRKL | KDM2B | PRKAR1A | RPTOR | NFE2L2 | HDAC4 | IRS2 | CDK8 |
| MSH2 | PRSS8 | CTNNA1 | AXL | MAP3K7 | FGFR2 | INHBA | FGFR4 | ESR1 |
| STAT4 | MAP2K2 | SMARCA1 | FBXO31 | NTRK2 | NTRK3 | CD22 | HIST1H2BC | EPHA5 |
| STAT5B | MKI67 | BLM | GID4 (C17orf39) | ERBB3 | PLCG2 | GPR124 | CBFB | NCSTN |
| HDAC1 | HDAC7 | FANCE | TGFBR2 | KEAP1 | PDCD1 | AURKA | FAM46C | INPP5D |
| DAXX | JUN | PTPN2 | MYCL (MYCL1) | PPP2R1A | DTX1 | FOXO3 | TOP1 | PDK1 |
| YY1AP1 | BCL2L2 | AKT2 | NKX2-1 | AURKB | MAGED1 | ZNF217 | NFKBIA | DNM2 |
| RNF43 | HIST1H2BK | IKZF2 | MAPK1 | HIST1H2AL | AR | SDHB |  |  |
| ACTB | APH1A | ASMTL | BCL11B | BTK | BTLA | CD79A | CHEK1 | DOT1L |
| DUSP2 | DUSP9 | EBF1 | ECT2L | ELP2 | EXOSC6 | FANCD2 | FANCF | FANCG |
| FANCL | FLYWCH1 | FOXL2 | FRS2 | GADD45B | GNA11 | GNAQ | GSK3B | HIST1H2BO |
| HSP90AA1 | IRF1 | MAP2K4 | MIB1 | MITF | MRE11A | MYO18A | NT5C2 | P2RY8 |
| PAG1 | PAK3 | PCBP1 | PRKDC | RASGEF1A | SDHD | SERP2 | SMAD2 | SMC3 |
| SOCS2 | SOX2 | SPOP | STAT5A | TMSB4XP8 (TMSL3) | TNFRSF17 | TSHR | U2AF2 | WDR90 |
| XBP1 | ZNF24 (ZSCAN3) |  |  |  |  |  |  |  |
| **Select introns** | | | | | | | | |
| ALK | BRAF | EPOR | ETV6 | IGK | JAK2 | NTRK1 | RAF1 | ROS1 |
| BCL2 | CCND1 | ETV1 | EWSR1 | IGL | KMT2A (MLL) | PDGFRA | RARA | TMPRSS2 |
| BCL6 | CRLF2 | ETV4 | FGFR2 | JAK1 | MYC | PDGFRB | RET | TRG |
| BCR | EGFR | ETV5 | IGH |  |  |  |  |  |
