# Supplementary material for: Distinct age-associated molecular profiles in acute myeloid leukemia defined by comprehensive clinical genomic profiling
Source: Oncotarget. 2018 May 29;9(41):26417–30. doi: 10.18632/oncotarget.25443 (PMC5995178; doi:10.18632/oncotarget.25443)
Supplement: Supplementary file 3 [file oncotarget-09-26417-s003.docx]

**Supplementary Table 1B: RNA transcripts targeted for capture in F1H assay**

| CCND1 | KMT2A (MLL) | WHSC1 (MMSET or NSD2) | BCL2 | IGH | BCL6 | MYC | ETV6 | RUNX1 |
| --- | --- | --- | --- | --- | --- | --- | --- | --- |
| ABL1 | CREBBP | CRLF2 | IGL | MLLT10 (AF10) | TCF3 (E2A) | MYH11 | NF1 | ALK |
| JAK2 | NOTCH1 | NSD1 | EP300 | CIITA | FOXP1 | NUP214 | TBL1XR1 | TP63 |
| FGFR3 | FUS | MECOM | IGK | RNF213 | BCR | KAT6A (MYST3) | PAX5 | PDGFRB |
| ARID1A | ASXL1 | BRAF | FGFR1 | PBX1 | PRDM1 | CCND3 | ERG | EWSR1 |
| MAFB | NPM1 | PPP1CB | ROS1 | TAL1 | ABL2 | BCL10 | BCL11A | BIRC3 |
| CIC | FLI1 | IKZF1 | JAK3 | JAZF1 | NUP98 | PDCD1LG2 (PDL2) | PDGFRA | RARA |
| ARHGAP26 (GRAF) | ATF1 | BCL7A | BCOR | CBFB | CDK6 | CTNNB1 | DUSP22 | EGFR |
| EIF4A2 | ETS1 | FOXO1 | HOXA9 | IRF4 | JAK1 | LPP | MKL1 | NTRK1 |
| PIM1 | PLAG1 | PML | POU2AF1 | RAF1 | RHOH | STAT6 | TFRC | TMPRSS2 |
| ABI1 | ACSL6 | AFF1 | AFF4 | ARHGEF12 | ARNT | ATG5 | ATIC | BCL11B |
| BCL3 | BCL9 | BTG1 | CAMTA1 | CARS | CBFA2T3 | CBL | CCND2 | CD274 (PDL1) |
| CDX2 | CHIC2 | CHN1 | CLP1 | CLTC | CLTCL1 | CNTRL (CEP110) | COL1A1 | CREB3L1 |
| CREB3L2 | CSF1 | DDIT3 | DDX10 | DDX6 | DEK | ELF4 | ELL | ELN |
| EML4 | EPOR | EPS15 | ERBB2 | ETV1 | ETV4 | ETV5 | FCGR2B | FCRL4 |
| FEV | FGFR1OP | FGFR2 | FNBP1 | FOXO3 | FOXO4 | FSTL3 | GAS7 | GLI1 |
| GMPS | GPHN | HERPUD1 | HEY1 | HIP1 | HIST1H4I | HLF | HMGA1 | HMGA2 |
| HOXA11 | HOXA13 | HOXA3 | HOXC11 | HOXC13 | HOXD11 | HOXD13 | HSP90AA1 | HSP90AB1 |
| IL21R | IL3 | ITK | KDSR | KIF5B | LASP1 | LCP1 | LMO1 | LMO2 |
| LYL1 | MAF | MALT1 | MDS2 | MLF1 | MLLT1 (ENL) | MLLT3 | MLLT4 (AF6) | MLLT6 |
| MN1 | MNX1 | MSI2 | MSN | MUC1 | MYB | MYH9 | NACA | NBEAP1 (BCL8) |
| NCOA2 | NDRG1 | NF2 | NFKB2 | NIN | NR4A3 | NTRK2 | NTRK3 | NUMA1 |
| NUTM2A | OMD | P2RY8 | PAFAH1B2 | PAX3 | PAX7 | PCM1 | PCSK7 | PDE4DIP |
| PDGFB | PER1 | PHF1 | PICALM | PRDM16 | PRRX1 | PSIP1 | PTCH1 | PTK7 |
| RABEP1 | RALGDS | RAP1GDS1 | RBM15 | RET | RPL22 | RPN1 | RUNX1T1 (ETO) | RUNX2 |
| SEC31A | 5-Sep | 6-Sep | 9-Sep | SET | SH3GL1 | SLC1A2 | SNX29 (RUNDC2A) | SRSF3 |
| SS18 | SSX1 | SSX2 | SSX4 | STL | SYK | TAF15 | TAL2 | TCL1A (TCL1) |
| TEC | TET1 | TFE3 | TFG | TFPT | TLX1 | TLX3 | TNFRSF11A | TOP1 |
| TPM3 | TPM4 | TRIM24 | TRIP11 | TTL | TYK2 | USP6 | WHSC1L1 | YPEL5 |
| ZBTB16 | ZMYM2 | ZNF384 | ZNF521 |  |  |  |  |  |
