# Supplementary material for: Distinct age-associated molecular profiles in acute myeloid leukemia defined by comprehensive clinical genomic profiling
Source: Oncotarget. 2018 May 29;9(41):26417–30. doi: 10.18632/oncotarget.25443 (PMC5995178; doi:10.18632/oncotarget.25443)

Supplemental Table 4: Tendencies of co-occurrence among the most commonly identified mutations in the cohort.


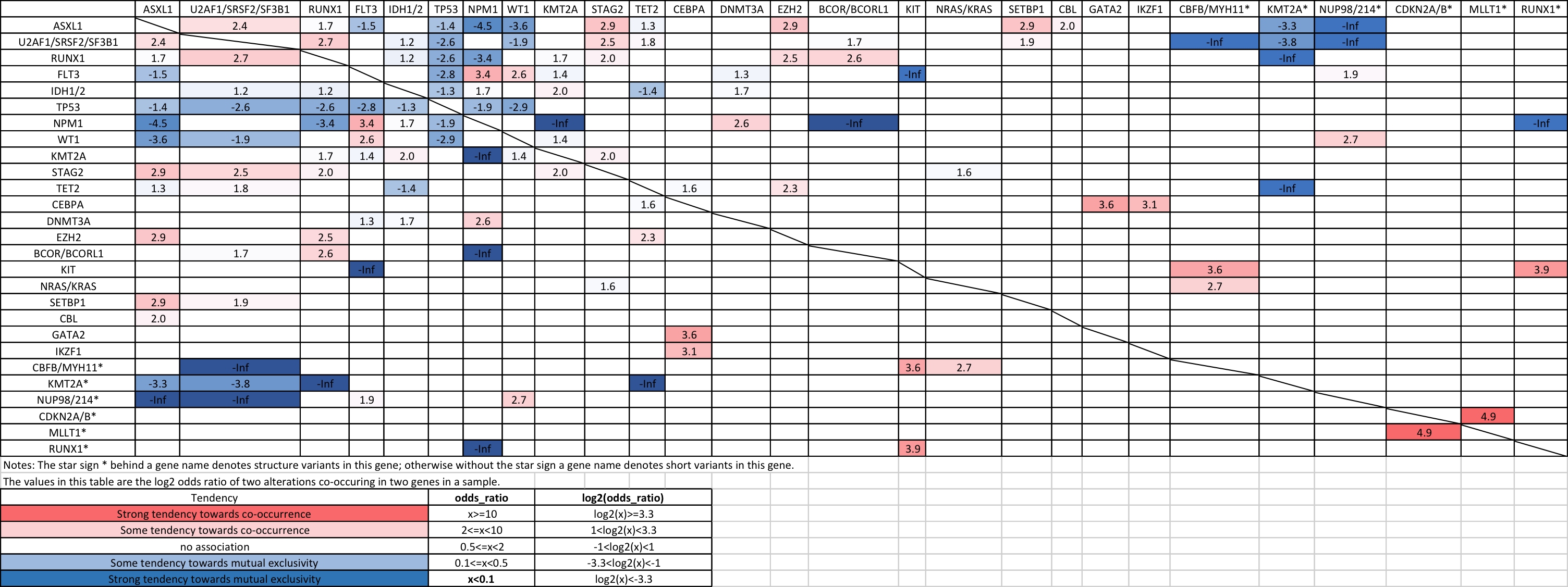

Supplement: Supplementary file 6 [file oncotarget-09-26417-s006.docx]
